# 2-(4-Chloro-2-nitro­phen­yl)-4-meth­oxy-9-phenyl­sulfonyl-9*H*-carbazole-3-carbaldehyde

**DOI:** 10.1107/S1600536814001809

**Published:** 2014-01-31

**Authors:** P. Narayanan, K. Sethusankar, Velu Saravanan, Arasambattu K. Mohanakrishnan

**Affiliations:** a Department of Physics, RKM Vivekananda College (Autonomous), Chennai 600 004, India; b Department of Organic Chemistry, University of Madras, Maraimalai campus, Chennai 600 025, India

## Abstract

In the sterically hindered title compound, C_26_H_17_ClN_2_O_6_S, the carbazole ring has a maximum deviation from planarity of 0.067 (4) Å for the C atom connected to the aldehyde group. The carbazole moiety forms a dihedral angle of 72.8 (1)° with the nitro-substituted benzene ring. The O atom of the meth­oxy group deviates by 0.186 (1) Å from the adjacent carbazole moiety. The phenyl­sulfonyl group forms intra­molecular C—H⋯O bonds between sulfone O atoms and the carbazole moiety, resulting in two *S*(6) rings. In the crystal, the nitrated benzene rings are linked *via* C—H⋯O inter­actions forming infinite *C*(7) chains along [100]. The crystal packing is also characterized by C—H⋯π inter­actions, which result in inversion dimers.

## Related literature   

For the biological activities and uses of carbazole derivatives, see: Itoigawa *et al.* (2000 ▶); Ramsewak *et al.*(1999 ▶). For their electronic properties and applications, see: Friend *et al.* (1999 ▶); Zhang *et al.* (2004 ▶). For related structures, see: Gopinath *et al.*(2013 ▶). For the Thorpe–Ingold effect, see: Bassindale (1984 ▶). For bond-length distortions, see: Allen *et al.* (1987 ▶). For graph-set notation: Bernstein *et al.* (1995 ▶).
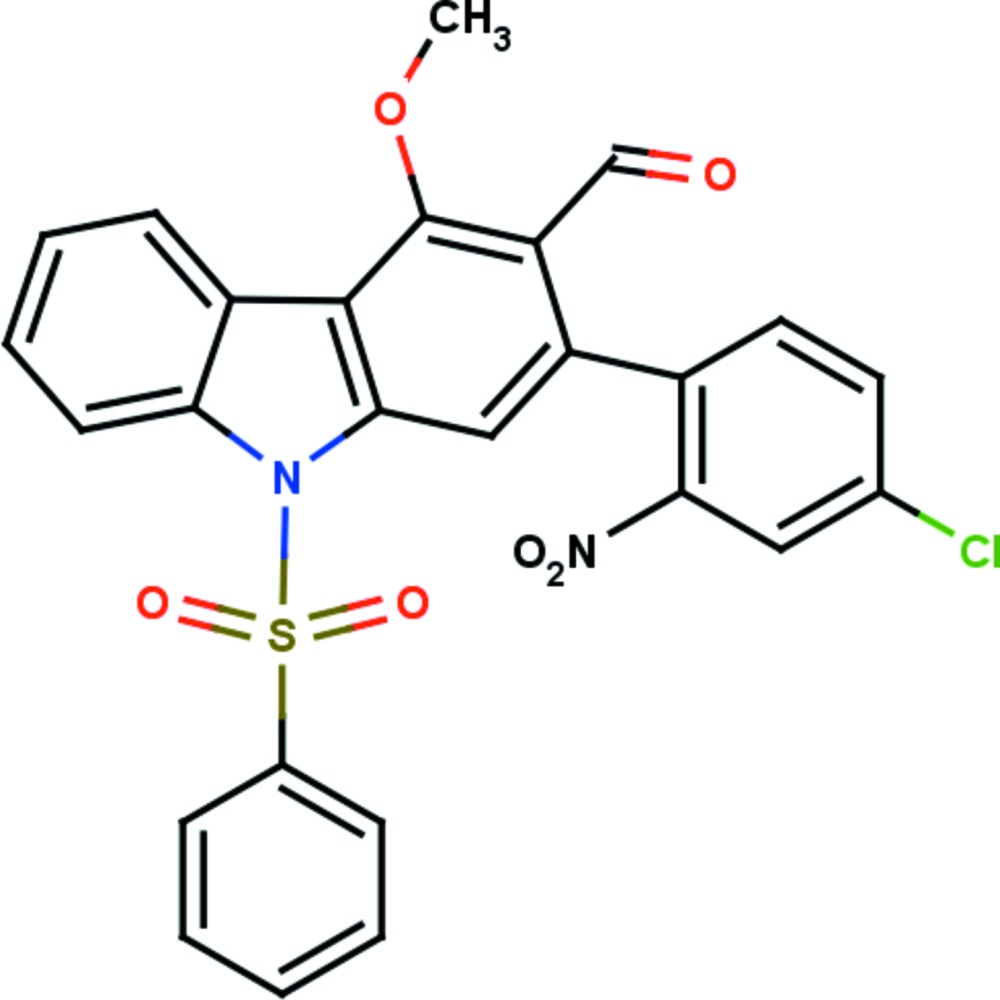


## Experimental   

### 

#### Crystal data  

C_26_H_17_ClN_2_O_6_S
*M* *_r_* = 520.94
Triclinic, 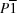

*a* = 8.1937 (5) Å
*b* = 11.6112 (8) Å
*c* = 12.4346 (9) Å
α = 93.886 (2)°
β = 93.952 (3)°
γ = 97.772 (2)°
*V* = 1165.80 (14) Å^3^
*Z* = 2
Mo *K*α radiation
μ = 0.30 mm^−1^
*T* = 296 K
0.25 × 0.25 × 0.20 mm

#### Data collection  

Bruker Kappa APEXII CCD diffractometer
Absorption correction: multi-scan (*SADABS*; Bruker, 2008 ▶) *T*_min_ = 0.928, *T*_max_ = 0.942
20355 measured reflections
4582 independent reflections
3911 reflections with *I* > 2σ(*I*)
*R*_int_ = 0.026

#### Refinement  

*R*[*F* ^2^ > 2σ(*F* ^2^)] = 0.056
*wR*(*F* ^2^) = 0.152
*S* = 1.04
4582 reflections
326 parameters
1 restraint
H-atom parameters constrained
Δρ_max_ = 1.00 e Å^−3^
Δρ_min_ = −0.55 e Å^−3^

### 

Data collection: *APEX2* (Bruker, 2008 ▶); cell refinement: *SAINT* (Bruker, 2008 ▶); data reduction: *SAINT*; program(s) used to solve structure: *SHELXS97* (Sheldrick, 2008 ▶); program(s) used to refine structure: *SHELXL97* (Sheldrick, 2008 ▶); molecular graphics: *ORTEP-3 for Windows* (Farrugia, 2012 ▶) and *Mercury* (Macrae *et al.,* 2008 ▶); software used to prepare material for publication: *SHELXL97* and *PLATON* (Spek, 2009 ▶).

## Supplementary Material

Crystal structure: contains datablock(s) global, I. DOI: 10.1107/S1600536814001809/ld2117sup1.cif

Structure factors: contains datablock(s) I. DOI: 10.1107/S1600536814001809/ld2117Isup2.hkl

Supporting information file. DOI: 10.1107/S1600536814001809/ld2117Isup3.cml

CCDC reference:

Additional supporting information:  crystallographic information; 3D view; checkCIF report

## Figures and Tables

**Table 1:** Hydrogen-bond geometry (Å, °) *Cg* is the centroid of the C19–C24 ring.

*D*—H⋯*A*	*D*—H	H⋯*A*	*D*⋯*A*	*D*—H⋯*A*
C2—H2⋯O1	0.93	2.37	2.955 (3)	121
C11—H11⋯O2	0.93	2.31	2.902 (3)	121
C15—H15⋯O3^i^	0.93	2.55	3.444 (4)	161
C4—H4⋯*Cg*1^ii^	0.93	2.94	3.715 (3)	142

Symmetry codes: (i) 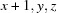; (ii) 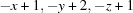.

**Fig. 1.**
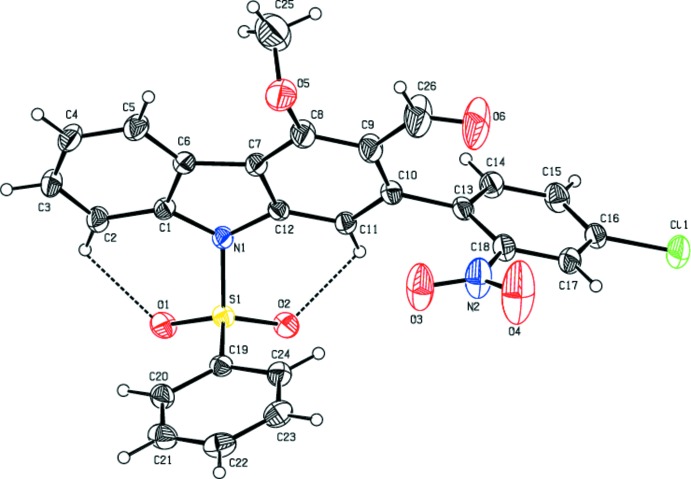
The molecular structure of the title compound with the atom numbering scheme, displacement ellipsoids are drawn at 30% probability level. H atoms are present as small spheres of arbitary radius. The intramolecular C—H···O hydrogen bonds, which are generate S(6) ring motifs, shown as a dashed lines (see Table 1 for details).

**Fig. 2.**
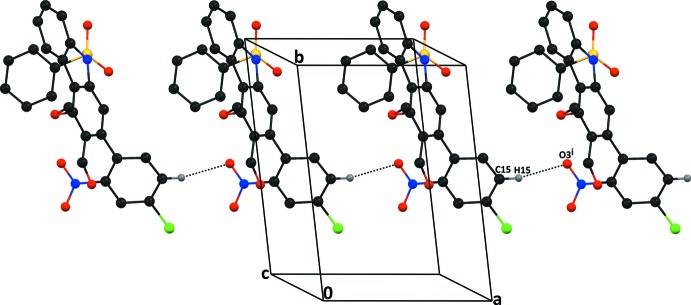
Part of the crystal packing of the title compound viewed down b axis. The dashed lines indicate C15—H15···O3i intermolecular hydrogen bonding, which generate C(7) infinite one dimensional chain running parallel to base vector [1 0 0]. Symmetry code: (i). 1 + x, y, z.

**Fig. 3.**
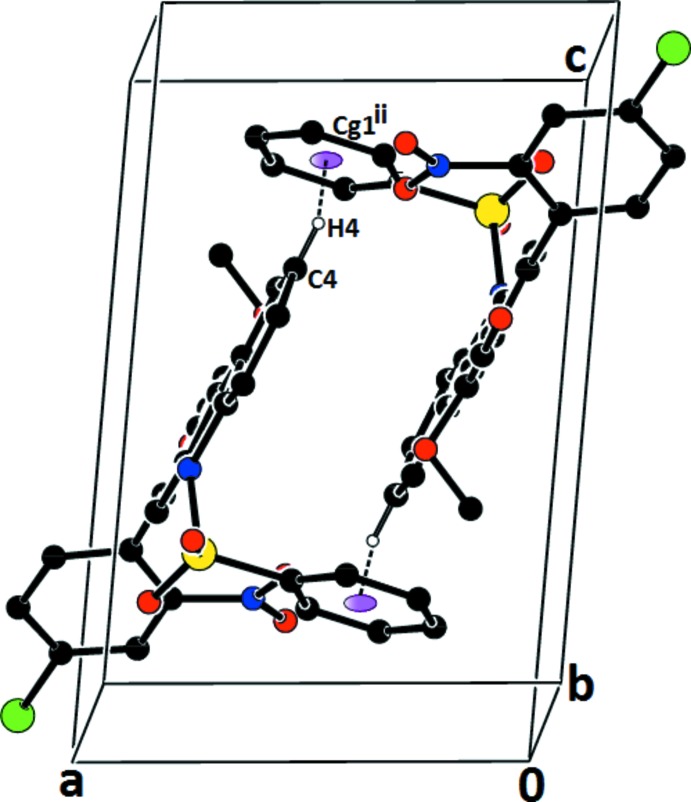
Part of the crystal packing of the title compound viewed down c axis. The dashed lines indicate C4—H4···Cg1ii interactions, where cg1 is the centre gravity of (C19–C24). Symmetry code: (ii). 1 - x, 2 - y, 1 - z.

## supplementary crystallographic information

### 1. Comment

Carbazole and its derivative have become quite attractive compounds owing to their applications in pharmacy and molecular electronics. It has been reported that carbazole derivatives exhibit various biological activities such as antitumor (Itoigawa, *et al.* 2000), anti-inflammatory and antimutagenic (Ramsewak, *et al.* 1999). Carbazole derivatives also exhibit electroactivity and luminenscence and are considered to be potential candidates for electronic applications such as colour displays, organic, semiconductors, laser and solar cells (Friend, *et al.* 1999; Zhang *et al.* 2004).

The title compound, C_26_H_17_ClN_2_O_6_S, comprises a carbazole ring system which is attached to a phenylsulfonyl ring, a chlorine substituted nitrophenyl ring, a methoxy group and a carbaldehyde group. The carbazole ring system is essentially planar with maximum deviation of 0.067 (4) Å for the carbon atom (C9), which is connected to the aldehyde group. The methoxy group oxygen atom(O5) and carbaldehyde group carbon atom (C26) are deviate from the carbazole ring system by 0.1860 (9) Å and 0.1767 (3) Å, respectively. The carbazole ring system make dihedral angles of 72.81 (11)° and 78.74 (11)° with the nitrophenyl ring(C13–C18) and sulfonyl sustituted phenylring(C19–C24), respectively.

The atom S1 has a distorted tetrahedral configuration. The widening of angle O2—S1—O1 [120.52 (12) °] and narrowing of angle N1—S1—C19 [104.80 (11)°] from the ideal tetrahedral value are attributed to the Thorpe-Ingold effect (Bassindale, 1984). As a result of electron-withdrawing character of the phenylsulfonyl group, the bond lengths N1–C1 = 1.429 (3) Å and N1–C12 = 1.418 (3) Å in the molecule are longer than the mean value of 1.355 (14) Å (Allen, *et al.* 1987). The sum of the bond angles around N1 [350.5°] indicate the sp2 hybridization. The chlorine atom Cl1 deviated by -0.0246 (9) Å from the phenyl ring (C13–C18).

The molecular structure is stabilized by C2—H2···O1, C11—H11···O2 intramolecular interactions formed by the sulfone oxygen atoms, which generate two *S*(6) ring motifs (Fig-1). In the crystal packing, molecules are linked *via* C15—H15···O3^i^ intermolecular hydrogen bonding, which generate *C*(7) infinite one dimensional chain running parallel to base vector [1 0 0]. The crystal packing is further stabilized by C4—H4···*Cg*1^ii^ intermolecular interaction, which results in centrosymmetric dimers (Bernstein, *et al.* 1995). The packing view of the title compound is shown in Fig-2 and Fig-3. Symmetry code: (i). 1 + *x*, *y*, *z*. (ii). 1 - *x*, 2 - *y*, 1 - *z*.

### 2. Experimental

A solution of 3-(bromomethyl)-2-(4-chloro-2-nitrophenyl)- 4-methoxy-9-(phenylsulfonyl)-9*H*-carbazole (1.50 g, 2.5 mmol) and bis(tetrabutylammonium) dichromate (2.63 g, 3.75 mmol) in dry CHCl_3_ (50 ml) was refluxed for 10 h. The susequent removal of solvent in vacuo followed by column chromatographic (silica gel; hexane-ethyl acetate, 4:1) purification afforded 9-(phenylsulfonyl)-2-(4-chloro- 2-nitrophenyl)-4-methoxy-9*H*-carbazole-3-carbaldehyde (1.08 g, 81%) as a colourless solid. Single crystal suitable for X-ray diffraction were prepared by slow evaporation of a solution of the title compound in chloroform (CHCl_3_) at room temperature. m.p. 499–501 K.

### 3. Refinement

The positions of hydrogen atoms were localized from the difference electron density maps and their distances were geometrically constrained. The hydrogen atoms bound to the C atoms are treated as riding atoms, with d(C—H)=0.93 and *U*iso(H) = 1.2Ueq(C) for aromatic and aldehyde group, d(C—H)=0.96 and *U*iso(H) =1.5*U*eq(C) for methyl group. The rotation angles for methyl group were optimized by least squares.

### Crystal data

**Table:**

C_26_H_17_ClN_2_O_6_S	*Z* = 2
*M**_r_* = 520.94	*F*(000) = 536
Triclinic, *P*1	*D*_x_ = 1.484 Mg m^−^^3^
Hall symbol: -P 1	Mo *K*α radiation, λ = 0.71073 Å
*a* = 8.1937 (5) Å	Cell parameters from 3911 reflections
*b* = 11.6112 (8) Å	θ = 1.7–26.0°
*c* = 12.4346 (9) Å	µ = 0.30 mm^−^^1^
α = 93.886 (2)°	*T* = 296 K
β = 93.952 (3)°	Block, colourless
γ = 97.772 (2)°	0.25 × 0.25 × 0.20 mm
*V* = 1165.80 (14) Å^3^	

### Data collection

**Table:**

Bruker Kappa APEXII CCD diffractometer	4582 independent reflections
Radiation source: fine-focus sealed tube	3911 reflections with *I* > 2σ(*I*)
Graphite monochromator	*R*_int_ = 0.026
ω & φ scans	θ_max_ = 26.0°, θ_min_ = 1.7°
Absorption correction: multi-scan (*SADABS*; Bruker, 2008)	*h* = −10→10
*T*_min_ = 0.928, *T*_max_ = 0.942	*k* = −14→14
20355 measured reflections	*l* = −15→15

### Refinement

**Table:**

Refinement on *F*^2^	Primary atom site location: structure-invariant direct methods
Least-squares matrix: full	Secondary atom site location: difference Fourier map
*R*[*F*^2^ > 2σ(*F*^2^)] = 0.056	Hydrogen site location: inferred from neighbouring sites
*wR*(*F*^2^) = 0.152	H-atom parameters constrained
*S* = 1.04	*w* = 1/[σ^2^(*F*_o_^2^) + (0.066*P*)^2^ + 1.5141*P*] where *P* = (*F*_o_^2^ + 2*F*_c_^2^)/3
4582 reflections	(Δ/σ)_max_ = 0.004
326 parameters	Δρ_max_ = 1.00 e Å^−^^3^
1 restraint	Δρ_min_ = −0.55 e Å^−^^3^

### Special details

**Table:**

Geometry. All e.s.d.'s (except the e.s.d. in the dihedral angle between two l.s. planes) are estimated using the full covariance matrix. The cell e.s.d.'s are taken into account individually in the estimation of e.s.d.'s in distances, angles and torsion angles; correlations between e.s.d.'s in cell parameters are only used when they are defined by crystal symmetry. An approximate (isotropic) treatment of cell e.s.d.'s is used for estimating e.s.d.'s involving l.s. planes.
Refinement. Refinement of *F*^2^ against ALL reflections. The weighted *R*-factor *wR* and goodness of fit *S* are based on *F*^2^, conventional *R*-factors *R* are based on *F*, with *F* set to zero for negative *F*^2^. The threshold expression of *F*^2^ > σ(*F*^2^) is used only for calculating *R*-factors(gt) *etc*. and is not relevant to the choice of reflections for refinement. *R*-factors based on *F*^2^ are statistically about twice as large as those based on *F*, and *R*- factors based on ALL data will be even larger.

### Fractional atomic coordinates and isotropic or equivalent isotropic displacement parameters (Å^2^)

**Table:**

	*x*	*y*	*z*	*U*_iso_*/*U*_eq_	
C1	0.7875 (3)	1.0463 (2)	0.4054 (2)	0.0331 (5)	
C2	0.7602 (4)	1.1615 (2)	0.4210 (2)	0.0434 (6)	
H2	0.7825	1.2139	0.3689	0.052*	
C3	0.6986 (4)	1.1949 (3)	0.5170 (3)	0.0484 (7)	
H3	0.6789	1.2715	0.5295	0.058*	
C4	0.6655 (4)	1.1179 (3)	0.5950 (2)	0.0519 (7)	
H4	0.6244	1.1434	0.6589	0.062*	
C5	0.6926 (4)	1.0039 (3)	0.5794 (2)	0.0502 (7)	
H5	0.6696	0.9521	0.6321	0.060*	
C6	0.7551 (3)	0.9672 (2)	0.4836 (2)	0.0372 (6)	
C7	0.7947 (3)	0.8559 (2)	0.4413 (2)	0.0378 (6)	
C8	0.7786 (5)	0.7452 (3)	0.4785 (3)	0.0550 (8)	
C9	0.8216 (5)	0.6511 (3)	0.4157 (3)	0.0541 (8)	
C10	0.8860 (3)	0.6700 (2)	0.3155 (2)	0.0384 (6)	
C11	0.9044 (3)	0.7800 (2)	0.2777 (2)	0.0344 (5)	
H11	0.9494	0.7928	0.2121	0.041*	
C12	0.8542 (3)	0.8706 (2)	0.3397 (2)	0.0313 (5)	
C13	0.9424 (3)	0.5760 (2)	0.2451 (2)	0.0379 (6)	
C14	1.1098 (4)	0.5799 (2)	0.2345 (3)	0.0457 (7)	
H14	1.1822	0.6399	0.2725	0.055*	
C15	1.1740 (4)	0.4983 (3)	0.1700 (3)	0.0500 (7)	
H15	1.2874	0.5035	0.1646	0.060*	
C16	1.0675 (4)	0.4088 (2)	0.1134 (2)	0.0424 (6)	
C17	0.9005 (4)	0.4010 (2)	0.1208 (2)	0.0451 (7)	
H17	0.8286	0.3403	0.0835	0.054*	
C18	0.8413 (3)	0.4851 (2)	0.1845 (3)	0.0434 (6)	
C19	0.6070 (3)	0.9713 (2)	0.15423 (19)	0.0343 (5)	
C20	0.4916 (4)	1.0486 (3)	0.1531 (2)	0.0440 (6)	
H20	0.5234	1.1277	0.1722	0.053*	
C21	0.3274 (4)	1.0045 (3)	0.1228 (3)	0.0544 (8)	
H21	0.2482	1.0548	0.1215	0.065*	
C22	0.2810 (4)	0.8885 (3)	0.0951 (2)	0.0535 (8)	
H22	0.1708	0.8605	0.0744	0.064*	
C23	0.3965 (4)	0.8121 (3)	0.0974 (3)	0.0516 (7)	
H23	0.3636	0.7329	0.0793	0.062*	
C24	0.5606 (4)	0.8532 (2)	0.1265 (2)	0.0419 (6)	
H24	0.6391	0.8023	0.1275	0.050*	
C25	0.8105 (7)	0.7460 (5)	0.6582 (5)	0.1012 (16)	
H25A	0.8853	0.6893	0.6552	0.152*	
H25B	0.7546	0.7410	0.7234	0.152*	
H25C	0.8712	0.8227	0.6572	0.152*	
C26	0.7960 (8)	0.5343 (3)	0.4572 (4)	0.1036 (19)	
H26	0.7534	0.5295	0.5245	0.124*	
N1	0.8522 (3)	0.98826 (17)	0.31621 (17)	0.0342 (5)	
N2	0.6608 (4)	0.4752 (3)	0.1854 (3)	0.0768 (10)	
O1	0.8401 (3)	1.14723 (16)	0.19275 (16)	0.0466 (5)	
O2	0.9140 (2)	0.95808 (17)	0.12450 (15)	0.0428 (5)	
O3	0.5979 (3)	0.5591 (3)	0.2093 (3)	0.1015 (12)	
O4	0.5802 (4)	0.3792 (3)	0.1634 (5)	0.154 (2)	
O5	0.6999 (4)	0.7255 (2)	0.5730 (2)	0.0744 (8)	
O6	0.8242 (7)	0.4466 (2)	0.4137 (3)	0.141 (2)	
S1	0.81663 (8)	1.02362 (5)	0.18874 (5)	0.03341 (18)	
Cl1	1.14424 (12)	0.30394 (8)	0.03361 (7)	0.0654 (3)	

### Atomic displacement parameters (Å^2^)

**Table:**

	*U*^11^	*U*^22^	*U*^33^	*U*^12^	*U*^13^	*U*^23^
C1	0.0332 (13)	0.0314 (12)	0.0331 (12)	0.0020 (10)	0.0000 (10)	−0.0008 (10)
C2	0.0545 (17)	0.0313 (13)	0.0444 (15)	0.0062 (12)	0.0046 (13)	0.0021 (11)
C3	0.0570 (18)	0.0357 (14)	0.0514 (17)	0.0095 (13)	0.0009 (14)	−0.0090 (12)
C4	0.065 (2)	0.0505 (17)	0.0398 (15)	0.0098 (15)	0.0102 (14)	−0.0083 (13)
C5	0.070 (2)	0.0471 (16)	0.0356 (14)	0.0105 (14)	0.0122 (14)	0.0028 (12)
C6	0.0422 (15)	0.0345 (13)	0.0344 (13)	0.0054 (11)	0.0000 (11)	0.0011 (10)
C7	0.0462 (15)	0.0342 (13)	0.0338 (13)	0.0078 (11)	0.0039 (11)	0.0030 (10)
C8	0.083 (2)	0.0384 (15)	0.0467 (15)	0.0128 (15)	0.0127 (14)	0.0115 (12)
C9	0.083 (2)	0.0346 (15)	0.0483 (17)	0.0134 (15)	0.0137 (16)	0.0107 (12)
C10	0.0424 (15)	0.0315 (13)	0.0416 (14)	0.0068 (11)	0.0011 (11)	0.0029 (11)
C11	0.0380 (14)	0.0317 (12)	0.0340 (13)	0.0063 (10)	0.0037 (10)	0.0029 (10)
C12	0.0321 (12)	0.0287 (12)	0.0330 (12)	0.0039 (9)	−0.0002 (10)	0.0051 (9)
C13	0.0414 (15)	0.0288 (12)	0.0437 (14)	0.0078 (10)	−0.0013 (11)	0.0041 (10)
C14	0.0398 (15)	0.0381 (14)	0.0562 (17)	0.0025 (12)	−0.0049 (13)	−0.0029 (12)
C15	0.0375 (15)	0.0467 (16)	0.067 (2)	0.0091 (12)	0.0048 (14)	0.0014 (14)
C16	0.0503 (17)	0.0363 (14)	0.0434 (15)	0.0147 (12)	0.0053 (12)	0.0043 (11)
C17	0.0487 (17)	0.0332 (14)	0.0514 (17)	0.0059 (12)	−0.0034 (13)	−0.0032 (12)
C18	0.0374 (15)	0.0356 (14)	0.0565 (17)	0.0063 (11)	0.0004 (12)	−0.0007 (12)
C19	0.0346 (13)	0.0411 (14)	0.0287 (12)	0.0080 (10)	0.0058 (10)	0.0048 (10)
C20	0.0466 (16)	0.0479 (16)	0.0408 (15)	0.0157 (13)	0.0076 (12)	0.0052 (12)
C21	0.0412 (17)	0.081 (2)	0.0471 (17)	0.0253 (16)	0.0075 (13)	0.0091 (16)
C22	0.0366 (16)	0.080 (2)	0.0419 (16)	0.0023 (15)	0.0028 (12)	0.0049 (15)
C23	0.0493 (18)	0.0541 (18)	0.0470 (17)	−0.0043 (14)	0.0021 (13)	−0.0020 (13)
C24	0.0422 (15)	0.0421 (15)	0.0416 (15)	0.0069 (12)	0.0037 (12)	0.0021 (11)
C25	0.092 (4)	0.086 (3)	0.125 (5)	0.022 (3)	−0.014 (3)	0.007 (3)
C26	0.204 (6)	0.043 (2)	0.076 (3)	0.030 (3)	0.057 (3)	0.0231 (19)
N1	0.0417 (12)	0.0282 (10)	0.0333 (11)	0.0057 (9)	0.0041 (9)	0.0042 (8)
N2	0.0408 (16)	0.0591 (18)	0.124 (3)	0.0025 (14)	0.0016 (17)	−0.0289 (19)
O1	0.0576 (13)	0.0341 (10)	0.0481 (11)	0.0005 (9)	0.0046 (9)	0.0139 (8)
O2	0.0412 (10)	0.0512 (11)	0.0402 (10)	0.0128 (9)	0.0144 (8)	0.0102 (8)
O3	0.0417 (14)	0.081 (2)	0.174 (3)	0.0164 (13)	−0.0013 (17)	−0.047 (2)
O4	0.0548 (19)	0.092 (3)	0.296 (6)	−0.0152 (17)	0.022 (3)	−0.077 (3)
O5	0.116 (2)	0.0623 (15)	0.0496 (13)	0.0163 (15)	0.0204 (14)	0.0153 (11)
O6	0.301 (6)	0.0359 (15)	0.105 (3)	0.040 (2)	0.090 (3)	0.0241 (15)
S1	0.0359 (3)	0.0318 (3)	0.0338 (3)	0.0044 (2)	0.0069 (2)	0.0082 (2)
Cl1	0.0788 (6)	0.0592 (5)	0.0625 (5)	0.0269 (4)	0.0160 (4)	−0.0087 (4)

### Geometric parameters (Å, º)

**Table:**

C1—C2	1.388 (4)	C16—C17	1.369 (4)
C1—C6	1.396 (4)	C16—Cl1	1.730 (3)
C1—N1	1.429 (3)	C17—C18	1.375 (4)
C2—C3	1.380 (4)	C17—H17	0.9300
C2—H2	0.9300	C18—N2	1.469 (4)
C3—C4	1.379 (4)	C19—C24	1.386 (4)
C3—H3	0.9300	C19—C20	1.389 (4)
C4—C5	1.375 (4)	C19—S1	1.756 (3)
C4—H4	0.9300	C20—C21	1.390 (4)
C5—C6	1.391 (4)	C20—H20	0.9300
C5—H5	0.9300	C21—C22	1.364 (5)
C6—C7	1.449 (4)	C21—H21	0.9300
C7—C8	1.389 (4)	C22—C23	1.382 (5)
C7—C12	1.398 (4)	C22—H22	0.9300
C8—O5	1.396 (4)	C23—C24	1.380 (4)
C8—C9	1.398 (4)	C23—H23	0.9300
C9—C10	1.406 (4)	C24—H24	0.9300
C9—C26	1.477 (5)	C25—O5	1.332 (6)
C10—C11	1.384 (4)	C25—H25A	0.9600
C10—C13	1.494 (4)	C25—H25B	0.9600
C11—C12	1.387 (3)	C25—H25C	0.9600
C11—H11	0.9300	C26—O6	1.178 (5)
C12—N1	1.418 (3)	C26—H26	0.9300
C13—C14	1.382 (4)	N1—S1	1.680 (2)
C13—C18	1.392 (4)	N2—O3	1.193 (4)
C14—C15	1.382 (4)	N2—O4	1.220 (4)
C14—H14	0.9300	O1—S1	1.4191 (19)
C15—C16	1.380 (4)	O2—S1	1.4226 (19)
C15—H15	0.9300		
			
C2—C1—C6	121.7 (2)	C15—C16—Cl1	120.2 (2)
C2—C1—N1	129.6 (2)	C16—C17—C18	118.5 (3)
C6—C1—N1	108.7 (2)	C16—C17—H17	120.8
C3—C2—C1	117.3 (3)	C18—C17—H17	120.8
C3—C2—H2	121.4	C17—C18—C13	123.6 (3)
C1—C2—H2	121.4	C17—C18—N2	116.1 (3)
C4—C3—C2	121.8 (3)	C13—C18—N2	120.3 (3)
C4—C3—H3	119.1	C24—C19—C20	121.3 (3)
C2—C3—H3	119.1	C24—C19—S1	118.9 (2)
C5—C4—C3	120.8 (3)	C20—C19—S1	119.8 (2)
C5—C4—H4	119.6	C19—C20—C21	118.2 (3)
C3—C4—H4	119.6	C19—C20—H20	120.9
C4—C5—C6	118.9 (3)	C21—C20—H20	120.9
C4—C5—H5	120.5	C22—C21—C20	120.8 (3)
C6—C5—H5	120.5	C22—C21—H21	119.6
C5—C6—C1	119.5 (3)	C20—C21—H21	119.6
C5—C6—C7	133.1 (3)	C21—C22—C23	120.6 (3)
C1—C6—C7	107.4 (2)	C21—C22—H22	119.7
C8—C7—C12	118.4 (2)	C23—C22—H22	119.7
C8—C7—C6	133.6 (3)	C24—C23—C22	120.0 (3)
C12—C7—C6	107.9 (2)	C24—C23—H23	120.0
C7—C8—O5	119.2 (3)	C22—C23—H23	120.0
C7—C8—C9	120.5 (3)	C23—C24—C19	119.1 (3)
O5—C8—C9	119.8 (3)	C23—C24—H24	120.5
C8—C9—C10	119.4 (3)	C19—C24—H24	120.5
C8—C9—C26	118.3 (3)	O5—C25—H25A	109.5
C10—C9—C26	122.2 (3)	O5—C25—H25B	109.5
C11—C10—C9	120.8 (3)	H25A—C25—H25B	109.5
C11—C10—C13	116.0 (2)	O5—C25—H25C	109.5
C9—C10—C13	123.2 (2)	H25A—C25—H25C	109.5
C10—C11—C12	118.5 (2)	H25B—C25—H25C	109.5
C10—C11—H11	120.8	O6—C26—C9	126.5 (4)
C12—C11—H11	120.8	O6—C26—H26	116.7
C11—C12—C7	122.3 (2)	C9—C26—H26	116.7
C11—C12—N1	129.1 (2)	C12—N1—C1	107.4 (2)
C7—C12—N1	108.6 (2)	C12—N1—S1	121.09 (17)
C14—C13—C18	115.5 (3)	C1—N1—S1	121.81 (17)
C14—C13—C10	118.3 (2)	O3—N2—O4	122.1 (3)
C18—C13—C10	126.2 (3)	O3—N2—C18	119.9 (3)
C13—C14—C15	122.7 (3)	O4—N2—C18	118.0 (3)
C13—C14—H14	118.7	C25—O5—C8	109.6 (4)
C15—C14—H14	118.7	O1—S1—O2	120.52 (12)
C16—C15—C14	119.1 (3)	O1—S1—N1	106.44 (11)
C16—C15—H15	120.5	O2—S1—N1	105.99 (11)
C14—C15—H15	120.5	O1—S1—C19	109.19 (13)
C17—C16—C15	120.6 (3)	O2—S1—C19	108.75 (12)
C17—C16—Cl1	119.2 (2)	N1—S1—C19	104.80 (11)
			
C6—C1—C2—C3	−0.4 (4)	C15—C16—C17—C18	0.8 (4)
N1—C1—C2—C3	−178.8 (3)	Cl1—C16—C17—C18	−180.0 (2)
C1—C2—C3—C4	0.2 (5)	C16—C17—C18—C13	−2.2 (5)
C2—C3—C4—C5	−0.2 (5)	C16—C17—C18—N2	177.4 (3)
C3—C4—C5—C6	0.4 (5)	C14—C13—C18—C17	2.3 (4)
C4—C5—C6—C1	−0.5 (5)	C10—C13—C18—C17	179.8 (3)
C4—C5—C6—C7	−178.8 (3)	C14—C13—C18—N2	−177.2 (3)
C2—C1—C6—C5	0.6 (4)	C10—C13—C18—N2	0.2 (5)
N1—C1—C6—C5	179.3 (3)	C24—C19—C20—C21	−0.3 (4)
C2—C1—C6—C7	179.2 (2)	S1—C19—C20—C21	178.4 (2)
N1—C1—C6—C7	−2.1 (3)	C19—C20—C21—C22	0.1 (4)
C5—C6—C7—C8	3.1 (6)	C20—C21—C22—C23	0.5 (5)
C1—C6—C7—C8	−175.4 (3)	C21—C22—C23—C24	−0.9 (5)
C5—C6—C7—C12	180.0 (3)	C22—C23—C24—C19	0.6 (4)
C1—C6—C7—C12	1.6 (3)	C20—C19—C24—C23	0.0 (4)
C12—C7—C8—O5	−171.8 (3)	S1—C19—C24—C23	−178.8 (2)
C6—C7—C8—O5	4.8 (6)	C8—C9—C26—O6	179.1 (6)
C12—C7—C8—C9	0.2 (5)	C10—C9—C26—O6	−0.3 (9)
C6—C7—C8—C9	176.9 (3)	C11—C12—N1—C1	179.2 (2)
C7—C8—C9—C10	1.8 (5)	C7—C12—N1—C1	−0.8 (3)
O5—C8—C9—C10	173.8 (3)	C11—C12—N1—S1	32.7 (4)
C7—C8—C9—C26	−177.6 (4)	C7—C12—N1—S1	−147.29 (19)
O5—C8—C9—C26	−5.6 (6)	C2—C1—N1—C12	−179.6 (3)
C8—C9—C10—C11	−1.2 (5)	C6—C1—N1—C12	1.8 (3)
C26—C9—C10—C11	178.3 (4)	C2—C1—N1—S1	−33.4 (4)
C8—C9—C10—C13	177.2 (3)	C6—C1—N1—S1	147.99 (19)
C26—C9—C10—C13	−3.4 (6)	C17—C18—N2—O3	−157.0 (4)
C9—C10—C11—C12	−1.5 (4)	C13—C18—N2—O3	22.6 (6)
C13—C10—C11—C12	−180.0 (2)	C17—C18—N2—O4	24.4 (6)
C10—C11—C12—C7	3.6 (4)	C13—C18—N2—O4	−155.9 (5)
C10—C11—C12—N1	−176.4 (2)	C7—C8—O5—C25	−89.5 (4)
C8—C7—C12—C11	−3.0 (4)	C9—C8—O5—C25	98.3 (4)
C6—C7—C12—C11	179.5 (2)	C12—N1—S1—O1	−174.10 (19)
C8—C7—C12—N1	177.0 (3)	C1—N1—S1—O1	44.2 (2)
C6—C7—C12—N1	−0.4 (3)	C12—N1—S1—O2	−44.7 (2)
C11—C10—C13—C14	69.0 (3)	C1—N1—S1—O2	173.67 (19)
C9—C10—C13—C14	−109.5 (3)	C12—N1—S1—C19	70.3 (2)
C11—C10—C13—C18	−108.5 (3)	C1—N1—S1—C19	−71.4 (2)
C9—C10—C13—C18	73.1 (4)	C24—C19—S1—O1	168.4 (2)
C18—C13—C14—C15	−1.2 (4)	C20—C19—S1—O1	−10.4 (2)
C10—C13—C14—C15	−178.9 (3)	C24—C19—S1—O2	35.1 (2)
C13—C14—C15—C16	−0.1 (5)	C20—C19—S1—O2	−143.7 (2)
C14—C15—C16—C17	0.2 (5)	C24—C19—S1—N1	−77.9 (2)
C14—C15—C16—Cl1	−179.0 (2)	C20—C19—S1—N1	103.3 (2)

### Hydrogen-bond geometry (Å, º)

Cg is the centroid of the C19–C24 ring.

**Table:**

*D*—H···*A*	*D*—H	H···*A*	*D*···*A*	*D*—H···*A*
C2—H2···O1	0.93	2.37	2.955 (3)	121
C11—H11···O2	0.93	2.31	2.902 (3)	121
C15—H15···O3^i^	0.93	2.55	3.444 (4)	161
C4—H4···*Cg*1^ii^	0.93	2.94	3.715 (3)	142

Symmetry codes: (i) *x*+1, *y*, *z*; (ii) −*x*+1, −*y*+2, −*z*+1.
